# Exploring Suicide Risk Factors in Schizophrenia: A Retrospective Study

**DOI:** 10.1192/j.eurpsy.2025.2256

**Published:** 2025-08-26

**Authors:** S. Ben Aissa, K. Razki, C. Najar, A. Larnaout, W. Melki

**Affiliations:** 1 Psychiatry, Razi hospital, Manouba, Tunisia

## Abstract

**Introduction:**

Suicide is a significant concern among individuals with schizophrenia, yet it often receives insufficient attention in clinical settings. Understanding the specific risk factors associated with suicide in this population is critical for implementing targeted and effective prevention strategies, enhancing patient care, and ultimately reducing this substantial risk.

**Objectives:**

To identify suicide risk factors within a population of patients with schizophrenia.

**Methods:**

This was a descriptive retrospective study conducted at the Psychiatry Department D of Razi Hospital in Tunisia. Clinical records of patients diagnosed with schizophrenia according to DSM-5 criteria, aged over 18 years, without substance-related disorders or somatic pathology explaining psychiatric symptoms, were reviewed. Patients were followed in the department for ten years (2013-2023) and identified as having at least one suicide attempt during their follow-up. Data collected included sociodemographic information (age, education level, residence, socioeconomic status, marital status, offspring, profession) and clinical details (number and nature of suicide attempts, clinical scores at the time of suicide attempt using PANSS and CDS scales, current antipsychotic treatment, family history of suicide and psychosis, and treatment adherence).

**Results:**

We collected data from 60 patients, with a mean age of 42 ± 12.02 years; 66% (n=40) were male. Regarding education and employment, 60% (n=36) had primary education, and 55% (n=33) were employed. 65% (n=39) were unmarried, and 80% (n=48) had a low socioeconomic status. The average number of suicide attempts per patient was 3.52 ± 1.02, with the most common methods being medication ingestion (60%) and strangulation (20%). Approximately 70% (n=42) of patients had a family history of mental disorders, and 40% (n=24) had a family history of suicide. During suicide attempts, mean scores on PANSS-positive, PANSS-negative, and CDS subscales were 25.4 ± 4.7, 18.6 ± 3.2, and 12.8 ± 2.5, respectively.

We found that male gender (p=0.03; OR=3.33; 95% CI [1.12 - 9.89]), low socioeconomic status (p=0.002; OR=2.25; 95% CI [1.04 - 4.86]), family history of suicide (p=0.04; OR=2.90; 95% CI [1.15 - 7.32]), high PANSS-positive scores (p=0.0019; OR=1.98; 95% CI [1.42 - 3.51]), and high CDS scores (p=0.005; OR=2.50; 95% CI [1.32 - 4.72]) were suicide risk factors in our study participants.

**Conclusions:**

The identified factors, including male gender, low socioeconomic status, family history of suicide, and elevated clinical symptomatology, highlight specific areas that warrant focused attention when evaluating and managing patients with schizophrenia.

**Disclosure of Interest:**

None Declared

